# Gestational age: comparing estimation methods and live births’ profile

**DOI:** 10.1590/1980-549720230016

**Published:** 2023-02-20

**Authors:** Eliana de Aquino Bonilha, Margarida Maria Tenório de Azevedo Lira, Marina de Freitas, Célia Maria Castex Aly, Patrícia Carla dos Santos, Denise Yoshie Niy, Carmen Simone Grilo Diniz

**Affiliations:** I Centro Universitário São Camilo – São Paulo (SP), Brazil.; II Universidade de São Paulo, School of Public Health, Grupo de Estudos Gênero, Evidências e Saúde – São Paulo (SP), Brazil.; III Pesquisa Dias Potenciais de Gravidez Perdidos – São Paulo (SP), Brazil.; IV Universidade de São Paulo, School of Public Health – São Paulo (SP), Brazil.

**Keywords:** Information systems, Birth certificates, Gestational age, Parturition, Sistema de informação, Declaração de nascido vivo, Idade gestacional, Parto

## Abstract

**Objective:**

To identify factors associated with the definition of the gestational age (GA) estimation method recorded in the live birth certificate (LBC), and to compare the results obtained according to the method in the city of São Paulo (CSP), between 2012 and 2019.

**Methods:**

Cross-sectional population-based study using the Live Birth Information System. Descriptive and comparative analysis was performed according to the GA estimation method, followed by a univariate and multivariate logistic regression model to identify the predictor variables of the method used.

**Results:**

The estimation of GA by the date of the last menstrual period (LMP) (39.9%) was lower than that obtained by other methods (OM) (60.1%) — physical examination and ultrasound, between 2012–2019. LMP registration in the LBC increased with the mother's age, it was higher among women who were white, more educated and with partners, in cesarean sections and with private funding. In the logistic regression, public funding was 2.33 times more likely than private funding to use OM. The proportion of preterm infants (<37 weeks) with GA by LMP was 26.5% higher than that obtained by OM. Median birth weight was higher among preterm infants with GA estimated by LMP.

**Conclusion:**

Prematurity was higher with the GA estimated by LMP in the CSP, which may indicate overestimation by this method. The source of funding was the most explanatory variable for defining the GA estimator method at the LBC. The results point to the need for caution when comparing the GA obtained by different methods.

## RESUMO

**Objetivo:**

Identificar fatores associados à definição do método estimador da idade gestacional (IG) registrado na declaração de nascido vivo (DNV) e comparar os resultados obtidos segundo método no município de São Paulo, entre 2012 e 2019.

**Métodos:**

Estudo transversal de base populacional utilizando o Sistema de Informações sobre Nascidos Vivos. Realizou-se análise descritiva e comparativa segundo método de estimativa da IG, seguida de modelo de regressão logística uni e multivariada para identificar as variáveis preditoras do método utilizado.

**Resultados:**

A estimativa da IG pela data da última menstruação (DUM) (39,9%) foi inferior à obtida por outros métodos (OM) (60,1%) — exame físico e ultrassonografia, entre 2012-2019. O registro da DUM na DNV aumentou com a idade da mãe, foi maior entre as brancas, mais escolarizadas e com companheiro, nas cesarianas e nos partos realizados com financiamento privado. Na regressão logística, o financiamento público apresentou chance 2,33 vezes maior que o privado para uso de OM. A proporção de prematuros (<37 semanas) com IG pela DUM foi 26,5% maior do que a obtida por OM. A mediana de peso ao nascer foi maior entre prematuros com IG estimada pela DUM.

**Conclusão:**

A prematuridade foi mais elevada com a IG estimada pela DUM no MSP, o que pode indicar superestimação por este método. A fonte de financiamento foi a variável mais explicativa para definição do método estimador da IG na DNV. Os resultados apontam a necessidade de cautela ao comparar a IG obtida por métodos diferentes.

## INTRODUCTION

“Term pregnancy” comprises the gestational age (GA) from 37 0/7 weeks to 41 6/7 weeks, and until recently was treated as a relatively homogeneous category. Due to this understanding, many cesarean sections were (and still are) scheduled before the onset of labor, from 37 weeks onward, when fetal development may still be incomplete^1^. Studies in the last decade have shown that live births at 37 to 38 weeks may have health outcomes more similar to those of late preterm infants (34–36 weeks), distancing themselves from the results of those born after 39 completed weeks^2–6^. For this reason, in 2013, the American College of Obstetricians and Gynecologists recommended a new classification for term newborns: “early-term” (37 0/7 to 38 6/7 weeks), “full-term” (39 0 /7 to 40 6/7 weeks) and “late-term” (41 0/7 to 41 6/7 weeks)^7^.

The most used methods to measure GA are ultrasonography (USG), the date of the last menstrual period (LMP), and the physical examination of the pregnant woman and the newborn, with varying accuracy and limitations^8–11^. USG, considered the gold standard for estimating GA, is more accurate depending on the date it is performed and is based on fetal measurements, varying according to the technology used and the date the exam was performed^12^. The World Health Organization recommends performing USG before 24 weeks of gestation to assess the health status of the pregnant woman and the fetus and to estimate the gestational age^13^. LMP, information provided by the pregnant woman, often considered as the first option as it does not depend on access to tests, may be inaccurate due to memory failures or irregular menstrual cycles^14^. GA can also be calculated by physical examination of the newborn or pregnant woman, which has been shown to be the least accurate method for estimating GA^10,15,16^.

Information about the duration of pregnancy is one of the main factors for predicting the health of newborns, as premature live births have a higher risk of morbidity and mortality^17,18^. Thus, monitoring the gestational age of live births from the SUS information systems can contribute to the formulation of public policies and actions to improve care. This is especially important when considering that Brazil is experiencing an epidemic of preterm and early-term births, largely related to the care provided to pregnant and parturient women^19^.

The Information System on Live Births (*Sistema de Informações sobre Nascidos Vivos* – Sinasc) was implemented by the Ministry of Health (MOH) in the 1990s, with the objective of collecting data on births throughout the national territory, with the Municipal Health Secretariats responsible for distributing, collecting, and processing the forms (Live Birth Certificate – LBC). Sinasc has coverage of almost 95% of information on live births in the country, although with significant variations between municipalities^20^. In the state of São Paulo, it is estimated that more than 99% of births are registered^20^. Specifically in São Paulo, the study showed high coverage, completeness, and reliability of information^21^.

The collection of information on GA, until 2010, only allowed the recording of GA ranges in weeks (less than 22 weeks; 22 to 27 weeks; 28 to 31; 32 to 36; 37 to 41; 42 and more weeks), in addition to not collecting the method used for its estimation. As of 2011, there was a change in the LBC, with the inclusion of new fields for GA disaggregated into weeks and for recording the method used to estimate it, allowing only one estimation method possible to be informed in the system^22,23^.

As there are several methods to estimate the GA, the MOH introduced two fields into the LBC, replacing the previous one (Figure S1). The first field (31) captures the date of the last menstrual period (LMP), the basis for calculating the GA in whole weeks, which is done later by the system itself. The other field (32) captures the number of whole weeks of gestation, to be filled in when LMP is ignored. This field also presents options to inform the method used in estimating the GA: “physical examination” or “another method”, the latter option being checked out in the case of ultrasonography^24^. The system accepts the “ignored” option both for the estimation method used and for the GA.

The GA information in the Sinasc database is only available in whole weeks, but it is also possible to calculate it in days for cases in which the LMP is registered. The use of the GA estimate in days implies a partial analysis of the Sinasc database, that is, only the records that had the LMP informed. However, studies have shown inconsistencies in estimating GA using LMP, with a bias toward increased prematurity, when compared with other estimation methods^16,25^.

This study aimed to identify the factors associated with the definition of the GA estimator method registered in the LBC and compare the results obtained according to the method in the city of São Paulo from 2012 to 2019.

## METHODS

This is a population-based cross-sectional study using the Sinasc database processed by the municipality of São Paulo from 2012 to 2019.

The field for recording the variable *gestational age in weeks* that appears in the Sinasc database is called SEMAGESTAC. When the LMP is registered in the system, the number of weeks is calculated automatically. If there is no LMP information, the GA in weeks is recorded from the baby's ultrasound or physical examination.

During the preparation of the database, it was observed that the system performs mathematical rounding when calculating the GA from the LMP, instead of considering only the number of complete weeks. Therefore, in this study, the GA was recalculated for records with LMP information, disregarding the fraction of days and considering only integer values^7,16,26^. To adjust this calculation, a new variable was created in the database, called SEMGESTCAL, based on the following formula: [(Date of birth of the baby — LMP)/7], considering weeks in whole numbers and disregarding fractions of days smaller than 7.

In the database set used in this study, the content of the field created to calculate the GA in weeks (SEMGESTCAL) is a hybrid of the weeks of gestation recalculated from the LMP and those recorded in SEMAGESTAC, when the estimation method was not the LMP.

Cases were then excluded according to the following criteria: GA <22 weeks or GA >45 weeks or GA ignored; birth weight <500 grams; maternal age <10 years or >49 years or unknown; deliveries that took place outside maternity hospitals or other types of health establishments; cases with missing information about type of delivery or type of pregnancy (Table 1).

**Table 1:** Number and proportion of live births, according to exclusion criteria. Municipality of São Paulo, 2012-2019.

Exclusion criteria by variable	Exclusions		Study population	
	n	%	n	%
			1,525,759	100.00
Maternal age <10 or >49 years or unknown	272	0.02	1,525,487	99.98
Type of pregnancy ignored	40	0.00	1,525,447	99.98
GA <22 or >45 weeks or ignored	4,388	0.29	1,521,059	99.69
Weight <500 grams	922	0.06	1,520,137	99.63
Non-hospital births	6,746	0.44	1,513,391	99.19
Type of delivery ignored	14	0.00	1,513,377	99.19
GA estimation method ignored	36,351	2.38		
Total	48,733	3.19	1,477,026	96.81

GA: gestational age.

For comparison purposes regarding the source of funding for childbirth, hospitals funded by the Unified Health System (*Sistema Único de Saúde* – SUS) were considered public, and hospitals funded by the supplementary health sector were considered private. This classification was carried out by the Sinasc team of the São Paulo Municipal Health Secretariat (*Secretaria Municipal de Saúde de São Paulo –* SMS/SP), based on the databases of the Hospital Information System (*Sistema de Informações Hospitalares –* SIH-SUS) and the National Register of Health Establishments (*Cadastro Nacional de Estabelecimentos de Saúde* – CNES-SUS) and inserted in the database of live births.

Initially, a bivariate descriptive analysis of the database was carried out with the characterization of the general profile of live births, followed by a comparative analysis of these profiles for the data set according to each GA estimation method. After this step, a univariate and multivariate logistic regression model was applied to estimate GA and to control confounding variables.

The methods for estimating GA were grouped into two categories for analysis purposes: date of last menstrual period (LMP) and other methods (OM), including physical examination (PE) and ultrasonography (USG), since, after comparing the GA by the different methods, great similarity was found between PE and USG (Table S1).

With the aim of verifying whether the source of financing for childbirth, type of delivery and maternal characteristics are predictors of the method used to calculate gestational age, a binary logistic regression was performed. In this case, the measures of association used were crude and adjusted odds ratios (OR), with 95% confidence intervals.

For this analysis, the LMP was considered the baseline category (b). For the selection of the dependent variables, the evolution of the categories during the period 2012–2019 was considered, with the variables most explanatory of the differences between the LB profiles being chosen, according to the GA estimation method. The dependent variables selected were: source of funding for childbirth (private [b] and public); type of delivery (cesarean [b] and vaginal); mother's age range (<20, 20 to 34 [b], and 35 years or more) and maternal education (no schooling, primary, secondary and higher education [b]); marital status (with a partner — married and in a stable union [b] — and without a partner — single, widowed, and separated).

The chi-square test was used to verify whether there were differences between the variables in each GA estimation method, considering a statistically significant difference if p-value <0.05.

Data processing and analysis were performed using SPSS software. The study was approved by the Research Ethics Committee of the School of Public Health of Universidade de São Paulo (CAAE: 98163018.2.0000.5421), on October 11^th^, 2018.

## RESULTS

Between 2012 and 2019, 1,525,759 LBC were processed at the CSP. In this study, 1,477,026 (96.8%) cases were considered, after performing the exclusions according to the criteria mentioned in the methodology, which represented 3.2% of the total LB (48,733) (Table 1).

In the analyzed period, the number of live births decreased by 5.8% in the city of São Paulo, the participation of SUS increased from 53.7% (2012) to 58.3% (2019) and the cesarean rate showed a decline of 6 .1% (Table S2).

**Figure 1:**
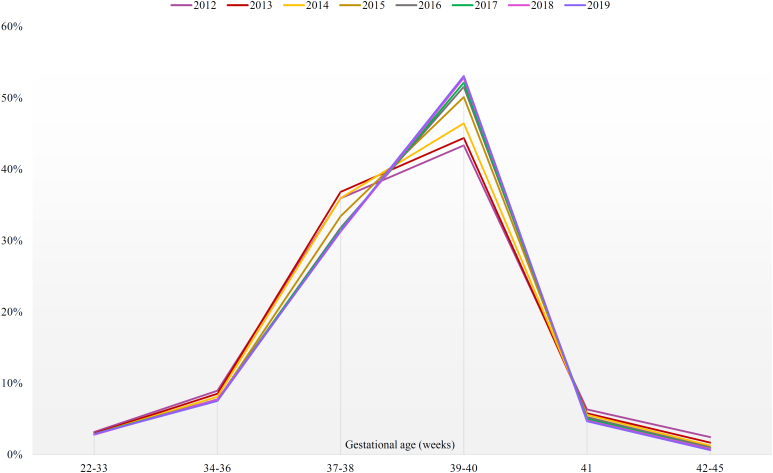
Proportion of live births, according to gestational age (in weeks) and year of birth. Municipality of São Paulo, 2012–2019.

With the exception of the group of live births considered full term (39–40 weeks of gestation), which increased by 22.5%, all other GA groups showed a reduction between 2012 and 2019. The decline in LMP information in the period is noteworthy, which went from 67.1% (2012) to 25.8% (2019) (Table S2 and Figure 1).

Comparing the LB profiles according to the method used to calculate the GA, it can be seen that the GA estimate by the LMP corresponded to 39.9% of the LB and by OM, to 60.1% in the analyzed period. The LMP record showed a higher proportion in women aged 20 to 34 and 35 years or older, among those with more schooling, among those with partners, in cesarean sections and in deliveries with private financing (Table 2) as well as among white subjects and among those who attended seven or more prenatal consultations (data not shown).

**Table 2:** Live births, by estimation method of gestational age and maternal, gestation and delivery characteristics. Municipality of São Paulo, 2012–2019.

		LMP		OM		Total	
		n	%	n	%	n	%
**Total**		**588,711**	**39.9**	**888,315**	**60.1**	**1,477,026**	**100,0**
Childbirth funding	Public	243,818	41.4	576,229	64.9	820,047	55.5
	Private	344,893	58.6	312,086	35.1	656,979	44.5
Type of delivery	Vaginal	204,817	34.8	438,667	49.4	643,484	43.6
	Cesarean section	383,894	65.2	449,648	50.6	833,542	56.4
Gestational age (weeks)	<34	18,819	3.2	23,726	2.7	42,545	2.9
	34 to 36	54,169	9.2	63,385	7.1	117,554	8.0
	37 to 38	215,729	36.6	279,266	31.4	494,995	33.5
	39 to 40	247,253	42.0	478,928	53.9	726,181	49.2
	41	37,603	6.4	40,831	4.6	78,434	5.3
	42 or+	15,138	2.6	2,179	0.2	17,317	1.2
Mother's age range (years)	10 to 19	50,542	8.6	113,495	12.8	164,037	11.1
	20 to 34	406,098	69.0	608,713	68.5	1,014,811	68.7
	35 or more	132,071	22.4	166,107	18.7	298,178	20.2
Mother's education	No education	457	0.1	1,006	0.1	1,463	0.1
	Elementary/Middle education	75,854	12.9	167,999	18.9	243,853	16.5
	High School	270,164	45.9	474,184	53.4	744,348	50.4
	Higher Education	241,604	41.0	244,566	27.5	486,170	32.9
Marital status	With partner	353,129	60.0	492,105	55.4	845,234	57.2
	No partner	234,634	39.9	394,963	44.5	629,597	42.6

P-value <0.001 for all variables. LMP: date of last menstrual period; OM: other methods (“physical examination” and “another method” (ultrasound) of live birth declaration). For Education and Marital Status, the ignored and uninformed did not appear.

The proportion of preterm infants (<37 weeks), when the GA was obtained using the LMP, was 12.4%, a value 26.5% higher than that estimated using OM (9.8%). Only in the full-term group (39 to 40 weeks) was the proportion of LB estimated by OM (53.9%) higher than that of LMP (42.0%) (Table 2).

**Figure 2:**
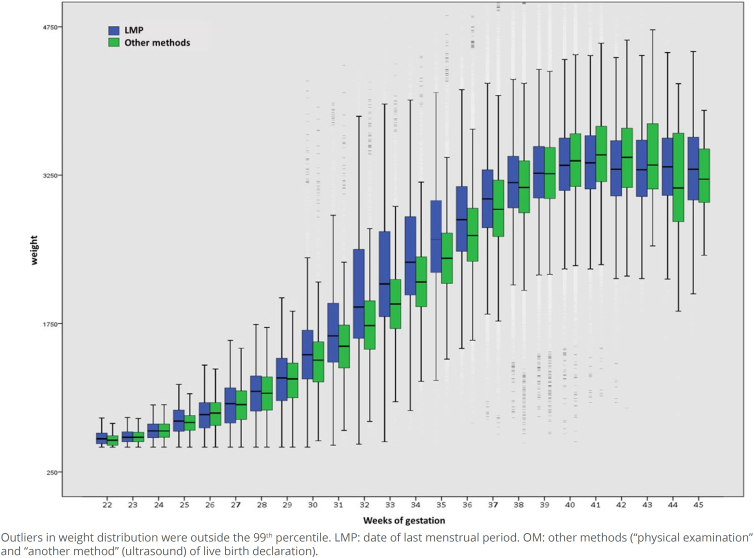
Distribution of live births, according to birth weight and gestational age, by estimation method. Municipality of São Paulo, 2012–2019.

The distribution of birth weight, according to weeks of gestation, stratified by the estimator method (LMP and OM), shows that the median weight was higher for live births with GA between 30 and 38 weeks of gestation, when estimated by LMP. The values were similar between the methods for babies born at 39 weeks and between 40 and 43 weeks, they were higher when GA was estimated by OM, with an inversion at weeks 44 and 45, when the LMP weight median exceeded that of OM (Figure 2).

In logistic regression, the selected variables showed a significant association with the use of other methods (p<0.001). In the adjusted model, public funding was 2.33 times more likely than private funding to use OM. This was also observed for vaginal delivery, with a 19% greater chance of registering OM than the cesarean section group. As for the variables maternal age and schooling, the chance in the group with OM records continued to be greater than the LMP, but with values below 10% for adolescent mothers (<20 years) and aged 35 years or more, and with high school and elementary education (Table 3).

**Table 3:** Univariate and multivariate logistic regression analysis for the gestational age estimation method, with the date of the last menstrual period being the baseline category, adjusted according to funding source, type of delivery, maternal age, and education. Municipality of São Paulo, 2012–2019.

		Gross OR	95%CI	p-value	Adjusted OR	95%CI	p-value
Childbirth funding source	Private	2.61	(2.59–2.63)	<0.001	2.33	(2.30–2.35)	<0.001
	Public (b)						
Type of delivery	Vaginal	1.83	(1.81–1.84)	<0.001	1.19	(1.18–1.20)	<0.001
	Cesarean section (b)						
Mother's age range (years)	10 to 19	1.50	(1.48–1.52)	<0.001	1.07	(1.05–1.08)	<0.001
	35 or more	0.84	(0.83–0.85)	<0.001	1.02	(1.02–1.03)	<0.001
	20 to 34 (b)						
Mother's education	No education	2.17	(1.95–2.43)	<0.001	1.06	(0.95–1.19)	0.279
	Elementary/Middle education	2.19	(2.17–2.21)	<0.001	1.06	(1.04–1.07)	<0.001
	High School	1.73	(1.72–1.75)	<0.001	1.05	(1.05–1.06)	<0.001
	Higher Education (b)						

b: baseline.

## DISCUSSION

The GA estimate by the date of the last menstrual period (39.9%) was lower than that obtained by other methods (60.1%), in the CSP, between 2012–2019. The proportion of preterm (<37 weeks) and early-term (37–38 weeks) births was higher when the GA estimate was calculated using the LMP. LMP registration in the LBC increased with the mother's age, it was higher among white mothers, those with more education and those with partners; in caesarean sections and deliveries carried out with private financing.

Regarding the higher proportions of prematurity observed when GA was estimated by LMP, it was found that the median birth weight was higher among preterm infants in this group, which may indicate overestimation of prematurity using this method. Similar results were observed in other studies that concluded that the LMP should not be the first method option to estimate the GA, when compared with the USG, as it can lead to over-enumeration of both prematurity and post-maturity^16,25,27^. Following the example of what was observed by Pereira et al.^15^, the results of the present study point to the need for caution when comparing the GA obtained by different methods, in addition to considering the source of financing for childbirth.

When applying the logistic regression technique, all the variables analyzed showed a significant association with the use of other methods (OM), with the source of funding being the most explanatory for defining the method registered in the LBC. The public network showed a greater chance, compared to the private network, of using OM, which was also observed for vaginal delivery, with a chance of about 20% greater than the cesarean section group, of registering OM. The odds in the group with an OM record continued to be greater than the LMP for adolescent mothers (<20 years old) and those aged 35 years old or older, and with lower education.

The increase in prematurity in the country has been pointed out for at least 15 years, and part of this increase may be related to the incorrect dating of the GA^28^ or the inaccuracy of the measurement^19^. Therefore, Sinasc can be improved to capture, in a more standardized and precise way, the relevant information for dating the pregnancy, including allowing the storage of more than one information about the GA and the method used for its calculation.

GA dating may present accuracy problems even with the use of early USG, as different measures and parameters are used to assess fetal development by sex or other individual variables, especially affecting the outcomes of late-preterm and early-term births^29–31^. In this sense, at the population level, increasing the granularity of information on GA from weeks to days can be of great value, to deepen the analysis of factors associated with the shortening of pregnancy and the negative outcomes on the health of mothers and babies^1^. In addition, research carried out nationwide recommends the combination of different methods to estimate GA, when available in the Sinasc database, such as LMP, physical examination, and ultrasound with the date it was performed^16^.

Therefore, there is a need to register the different GA estimation methods in the LBC, as well as the date on which the USG was performed, in addition to adapting Sinasc to include all options in the database, allowing the identification of inconsistencies and distortions in the measures, with a view to improving monitoring to support the implementation of good practices in labor and birth care.

The study has some limitations, such as those inherent to the use of a secondary database^32^. The inclusion of multiple pregnancies is also an aspect that makes comparison with other studies difficult.

Among the strengths of the study, the classification of the source of financing for deliveries stands out, a highly explanatory variable for the results. Additionally, the Sinasc database used has high coverage, completeness, and reliability, due to the different strategies for improving the quality of information developed by the CSP^21^.
